# Antibacterial Activity of *Pseudonocardia* sp. JB05, a Rare Salty Soil Actinomycete against *Staphylococcus aureus*

**DOI:** 10.1155/2014/182945

**Published:** 2014-08-14

**Authors:** Nesa Jafari, Reza Behroozi, Davoud Farajzadeh, Mohammad Farsi, Kambiz Akbari-Noghabi

**Affiliations:** ^1^Department of Molecular Genetics, National Institute of Genetic Engineering and Biotechnology, Tehran, Iran; ^2^Department of Plant Biotechnology, Buali-Sina University of Hamedan, Iran; ^3^Biotechnology Research Center, Biomedicine Institute, Tabriz University of Medical Sciences, Tabriz, Iran; ^4^Department of Cellular and Molecular Biology, Faculty of Biological Sciences, Azarbaijan Shahid Madani University, Tabriz, Iran; ^5^Department of Plant Biotechnology, Ferdowsi University of Mashhad, Mashhad, Iran

## Abstract

*Staphylococcus aureus* is a Gram-positive bacterium that causes many harmful and life-threatening diseases. Some strains of this bacterium are resistant to available antibiotics. This study was designed to evaluate the ability of indigenous actinomycetes to produce antibacterial compounds against *S. aureus* and characterize the structure of the resultant antibacterial compounds. Therefore, a slightly modified agar well diffusion method was used to determine the antibacterial activity of actinomycete isolates against the test microorganisms. The bacterial extracts with antibacterial activity were fractionated by silica gel and G-25 sephadex column chromatography. Also, the active fractions were analyzed by thin layer chromatography. Finally, the partial structure of the resultant antibacterial compound was characterized by Fourier transform infrared spectroscopy. One of the isolates, which had a broad spectrum and high antibacterial activity, was designated as *Pseudonocardia* sp. JB05, based on the results of biochemical and 16S rDNA gene sequence analysis. Minimum inhibitory concentration for this bacterium was 40 AU mL^−1^ against *S. aureus*. The antibacterial activity of this bacterium was stable after autoclaving, 10% SDS, boiling, and proteinase K. Thin layer chromatography, using anthrone reagent, showed the presence of carbohydrates in the purified antibacterial compound. Finally, FT-IR spectrum of the active compound illustrated hydroxyl groups, hydrocarbon skeleton, and double bond of polygenic compounds in its structure. To the best of our knowledge, this is the first report describing the efficient antibacterial activity by a local strain of *Pseudonocardia.* The results presented in this work, although at the initial stage in bioactive product characterization, will possibly contribute toward the *Pseudonocardia* scale-up for the production and identification of the antibacterial compounds.

## 1. Introduction

*Staphylococcus aureus *(*S. aureus*) is a facultative anaerobic, Gram-positive coccus. This bacterium is the most common cause of a wide variety of illnesses, such as impetigo, pimples, boils (furuncles), cellulitis folliculitis, carbuncles, scalded skin syndrome, and abscesses, as well as life-threatening diseases, such as pneumonia, osteomyelitis, meningitis, endocarditis, toxic shock syndrome (TSS), bacteremia, and sepsis. It can affect almost any parts of the body, including skin, connective tissue, respiratory, bone, joint, and endovascular regions. One estimate indicated that nearly 500,000 patients are admitted to American hospitals annually due to staphylococcal infection [1]. Penicillin is a common medication used to treat an* S. aureus* infection. However, the problem of penicillin resistance is extremely pronounced in most countries, where first-line therapy is most commonly a penicillinase-resistant *β*-lactam antibiotic such as oxacillin or flucloxacillin [2]. Soil bacteria belonging to actinomycetes group are one of the best sources of bioactive natural compounds used as antibiotics, pesticides, pharmaceuticals, herbicides, antiparasitics, and enzymes [3, 4]. About 13,000 biologically active secondary metabolites have been discovered from actinomycetes, and from which 70% have already been purified [5]. Within actinomycetes,* Streptomyces* is the most investigated genus because of either commercial interests or dominance of the genus on dilution plates and its facility of isolation [6].

It has been found that among actinomycetes the frequencies of isolation for* Streptomyces, Actinoplanes, Actinomadura, Microbispora, Micromonospora, Nocardia, Pseudonocardia, Streptosporangium, Thermoactinomyce, *and* Thermomonospora* were 95.3, 0.2, 0.1, 0.18, 1.4, 1.98, 0.06, 0.10, and 0.14%, respectively.

Non-Streptomycete actinomycetes (NSA) are a group of actinomycetes that were reviewed by El-Tarabily and Sivasithamparam [6]. H. A. Lechevalier and M. P. Lechevalier (1967) isolated 5000 actinomycetes from the soil and found that the genera of NSA, which were evaluated as rare,* Thermomonospora, Actinoplanes, Microbispora, Thermoactinomyces*,* Streptosporangium, Micropolyspora, Pseudonocardia, and Microellobosporia*, form less than 0.2% of the total isolates [7].

The family of Pseudonocardiaceae contains seven genera (*Actinopolyspora, Amycolata, Amycolatopsis, Kibdelosporangium, Pseudonocardia, Saccharomonospora, *and* Saccharopolyspora*) [8], several of them with industrial interest in bioconversion processes as antibiotic producers, including erythromycin, vancomycin, and rifamycin [9]. Li et al. (2011) reported the production of three new diazaanthraquinone derivatives by the strain SCSIO 01299, a marine actinomycete member of the genus* Pseudonocardia*, isolated from deep-sea sediment of the South China Sea. They found that some of these compounds exhibited potent cytotoxic activities against three tumor cell lines (SF-268, MCF-7, and NCI-H460) with IC50 values between 0.01 and 0.21 *μ*M and also showed antibacterial activities against* Staphylococcus aureus* ATCC 29213,* Enterococcus faecalis *ATCC 29212, and* Bacillus thuringensis *SCSIO BT01, with minimum inhibitory concentration (MIC) values of 1–4 *μ*g mL^−1^ [10].

As it was referred to earlier, actinomycetes produce many useful antibiotics against a large number of pathogenic bacteria. In the last decade, the screening for new secondary metabolites with antibacterial activity has also focused on minor groups of actinomycetes, including species that are difficult to isolate and culture, and those that grow under extreme conditions (i.e., alkaline and acidic conditions) [11]. Therefore, this study was designed to evaluate the ability of indigenous actinomycete strain(s) to produce antibacterial compound(s) against* S. aureus* in order to identify potent antibacterial compound(s).

## 2. Materials and Methods

### 2.1. Bacterial Strains and Growth Conditions

For the isolation of actinomycetes, twenty-three soil samples were collected from different saline and alkaline affected soils located in the vicinity of Hoze-Soltan, Qom, Iran.

Four different media, Water Yeast Extract Agar (WYE): 0.25 g of yeast extract, 0.5 g of K_2_HPO_4_, and 18 g of agar per liter of tap water, Starch Casein Agar (SCA): Soluble starch, 10.0 g; K_2_HPO_4_, 2.0 g; KNO_3_, 2.0 g; NaCl, 2.0 g; Casein, 0.3 g; MgSO_4_ *·*7H_2_O, 0.05 g; CaCO_3_, 0.02 g; FeSO_4_ *·*7H_2_O, 0.01 g, and agar 15.0 g, pH 8, Czapek Dox Agar (Sucrose, 30.0 g; NaNO_3_ 3.0 g; K_2_HPO_4_ 1.0 g; KCl 0.5 g; MgSO_4_ *·*7H_2_O, 0.5 g; FeSO_4_ *·*7H_2_O, 0.01 g; and agar 15.0 g; pH 7), and ISP_2_ (Malt extract, 10.0 g; Yeast extract, 4.0 g; Glucose, 4.0 g, and agar 20.0 g; pH 7) were used for identification, isolation, and preservation of actinomycetes. All chemical reagents and solvents were provided from Merck, Germany.

To provide antibacterial compounds secreted out into the culture medium, actinomycete isolates were cultured into ISP_2_ broth medium and incubated on a rotary shaker at 160 rpm for 14 days at 30°C.

Test bacteria, including* Staphylococcus aureus, Bacillus subtilis, B. pusteurii, Escherichia coli, Klebsiella *sp*., Pseudomonas aeruginosae, Xanthomonas *sp., and* Acetinobacter *sp*.,* provided from the Persian Type Culture Collection of the Iranian Research Organization for Science and Technology (IROST) (http://portal.irost.org/persian/PTCC/), were grown overnight in Luria-Bertani (LB) medium at 30°C.

The preliminary identification of isolates were carried out based on the result of biochemical conventional tests analysis [12]. Morphology and Gram stain were also determined by the use of a light microscope (1,000x) (Zeiss, Argentina S.A).

### 2.2. Antibacterial Activity Assay

A slightly modified agar well diffusion method [13] was adopted to determine the antibacterial activity of twenty-six actinomycete isolates against the test microorganisms with three replicates. Briefly, eight millimeter wells were made in Muller Hinton agar plates and inoculated with the test microorganisms. The same concentration of the diluted extracts of each actinomycete isolates then was poured into the wells. After 15-, 24- and 48-hour incubation at 28°C, the antibacterial activity of isolates was evaluated by measuring the diameter of the clear inhibition zones around the wells. The minimum inhibitory concentrations (MICs) were determined using the microplate dilution method.

The protein concentration of cell lysates was determined by the Bradford's method [14].

### 2.3. Preparation of DNA and Amplification of 16S rDNA Gene

DNA was prepared using a DNA extraction kit (QIAamp DNA Mini Kit, USA) and 16S rRNA encoding gene, amplified using primers S-C-Act-235-S-20 (5′-CGCGGCCTATCAGCTTGTTG-3′) and S-C-Act-878-A-19 (5′-CCGTACTCCCCAGGCGGGG-3′) [15]. PCR reactions were carried out in 25 *μ*L reaction mixtures containing PCR buffer 10X (2.5 *μ*L), 10 mM dNTPs (0.5 *μ*L), 1.5 mM MgCl_2_ (0.75 *μ*L), 2 *μ*M of each primer, 1 unit of* Taq* DNA polymerase, and 50 ng of genomic DNA. All the PCR reagents were purchased from Fermentas (St leon Rot, Lithuania). PCR was conducted in a DNA Thermal Cycler (Techne Flexigen, Minneapolis, MN, USA) under the following conditions: 3 min initial denaturation at 94°C, 30 cycles of 40 s denaturation at 94°C, primer annealing at 58°C for 40 s, and 45 s of elongation at 72°C, followed by a final extension at 72°C for 10 min. PCR products were loaded on 0.8% agarose gel to ensure that the size range of amplified 16S rDNA fragments were between 500 and 625 bp. 16S rDNA genes of the antibacterial-producing bacteria were sequenced and the nucleotide sequences were deposited in GenBank database. The 16S rDNA sequences were used to search the GenBank database using a nucleotide blast algorithm (http://blast.ncbi.nlm.nih.gov/) to display the closest matches to the 16S rDNA sequences for known species. Sequences were aligned with illustrative actinomycete 16S rDNA sequences and a phylogenetic tree was constructed by the neighbor-joining method, using the Molecular Evolutionary Genetics Analysis (MEGA) software version 5.0 [16, 17]. One thousand (1,000) bootstrap replications were used to evaluate the branched supporting values.

### 2.4. Purification and Partial Characterization of Antibacterial Compounds

The following procedure was used to extract and isolate antibacterial compound(s) from* Pseudonocardia *sp. JB05. Briefly, 50 mL of supernatant of bacterial culture was mixed with the same volume of absolute ethanol (Merck, Germany) and centrifuged at 2500 g to remove any bacterial cells. The cell free supernatant was concentrated (20X) using a vacuum centrifugation (Speed Vac AES 1010, Savant). The concentrated supernatant was dissolved with 1 mL of distilled water (dH_2_O), gently vortexed, and extracted sequentially with dH_2_O, n-hexane and/or ethyl acetate [18].

The antibacterial activity of the extracts was checked using an agar-well diffusion method against* S. aureus *as above mentioned [13].

The extracts with antibacterial activity were fractionated by silica gel column chromatography (610 × 16 mm) in dH_2_O (0.5 mL min^−1^). The active fractions were pooled and loaded onto G25 column chromatography and analyzed by thin layer chromatography (TLC) on silica gel plates (SiO_2_, Merck) with acetonitrile (ACN): methanol (MeOH): dH_2_O (6 : 1 : 3, v/v/v) [18]. The anthrone reagent (0.1 g anthrone, 50 mL pure H_2_SO_4_, and 5 mL dH_2_O) was sprayed onto the silica plate to detect the soluble sugars. Anthrone is a tricyclic aromatic ketone that is used for the colorimetric determination of carbohydrates [19].

The partial structure of the resultant antibacterial compounds was then characterized by Fourier transform infrared spectroscopy (FTIR). The spectra were also scanned in the range of 400 to 4000 cm^−1^ and plotted as intensity versus wavelength [20, 21].

### 2.5. Stability of Antimicrobial Compounds

The stability of antibacterial compounds was tested by treating them with proteinase K, 10% sodium dodecyl sulfate (SDS), and boiling. An experiment based on a completely randomized factorial design with three replicates was performed and the effect of two factors A: four cell free supernatants (A_1:_ number 012-1, A_2_: number 012-2, A_3_: number 010-31, and A_4:_ number 025-26) and B: destructive agents (B_1_: proteinase K, B_2_: 10% SDS and B_3_: boiling) were investigated. The bacterial effective extracts were exposed to destructive agents for enough time and their antibacterial activities were then examined against* S. aureus* as described earlier; all data were analyzed by JMP7 software (SAS Institute Inc). Mean comparison was carried out by Tukey's LSD method.

## 3. Results and Discussion

### 3.1. Selection and Identification of Actinomycetes

Sporulation of actinomycetes makes them easy to identify on agar plates. From among four media (WYE, SCA, CzapekDox Agar, and ISP_2_), WYE was the best medium for the isolation of actinomycetes. In general, this medium is poor in organic carbon, which effectively controlled fungal and eubacterial growth and thus helping in the isolation of the slower growing actinomycetes [22]. Since WYE and YCED media were especially effective for the isolation of actinomycetes, they were used predominantly [23]. In this research, sixty actinomycetes were isolated from different parts of salty and alkaline soil samples collected from Hoze-soltan in Qom, Iran. The isolates were identified based on the result of morphological and biochemical conventional tests analysis. Accordingly, the isolate number 010-31 was designated as* Pseudonocardia *sp. JB05 (Table 1).

### 3.2. Antibacterial Assay

**Figure 1:**
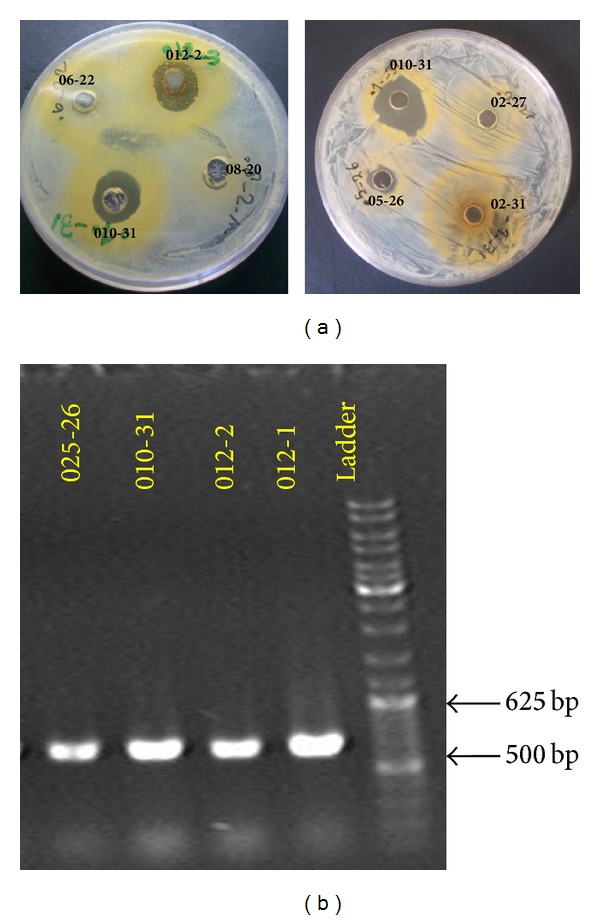
Screening of actinomycete isolates based on (a) their antibacterial activity against* S. aureus* and (b) their 16S rDNA gene fragments. From right to left: gel electrophoresis of 1 kb DNA ladder and the PCR products of 16S rDNA gene fragments. Numbers shown above each lane or on each plate are the numbers of the actinomycete isolates.

Antibacterial activity assay was performed using well diffusion method. Among the sixty isolated actinomycetes, only four strains (number 012-1, number 012-2, number 010-31, and number 025-26) showed antibacterial activity against at least one test microorganisms (Figure 1(a)). Strain number 010-31 had the highest inhibitory effect on the growth of all tested pathogenic bacteria, especially on* S. aureus *(Table 1). pH and temperature ranges for the growth of isolate number 010-31 were 6–10 and 27–32°C, respectively, with an optimal growth on pH 8 and 30°C, respectively. It was also capable to grow in WYE medium containing 5% w/v NaCl.

### 3.3. Molecular Analysis and Phylogenic Studies

**Figure 2:**
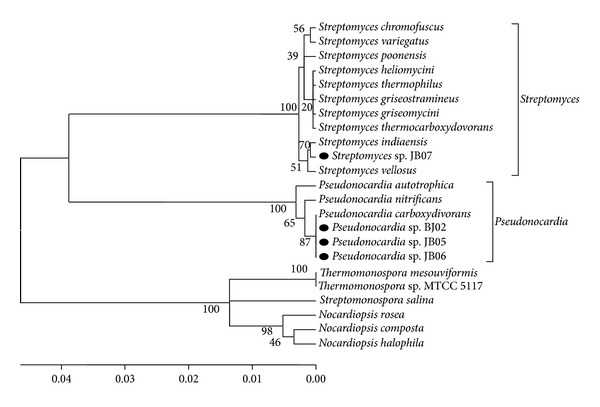
Phylogenetic tree of the 16S rDNA nucleotide sequences. Numbers above branches represent bootstrap values (1000 replicates) using neighbor joining.

Actinomycetes specific primers were used to amplify 16S rDNA gene (500–625 bp). These primers that were earlier used by Stach [15], these specific primers, can easily identify actinomycetes from other bacteria (Figure 1(b)). The 16S rDNA sequences of the four antibacterial-producing isolates (number 012-1, number 012-2, number 010-31, and number 025-26) were blasted using megablast tool of GenBank (http://www.ncbi.nlm.nih.gov/) and were deposited in the NCBI database as* Streptomyces* sp. JB07 (HQ896734),* Pseudonocardia* sp. JB06 (HQ896733),* Pseudonocardia* sp. JB05 (HQ896675), and* Pseudonocardia* sp. JB02 (HQ398191). Analysis of phylogenetic tree by using 16S rDNA sequences had led to the formation of three main clades, including* Streptomyces*,* Pseudonocardia,* and* Nocardiopsis* (Figure 2). These isolated bacteria belong to the two main families of actinomycetes; Streptomycetaceae, with a frequency of 95.3%, and* Pseudonocardiaceae*, with a frequency of 0.06% [24].

### 3.4. Purification and Partial Characterization of Antibacterial Compound

**Figure 3:**
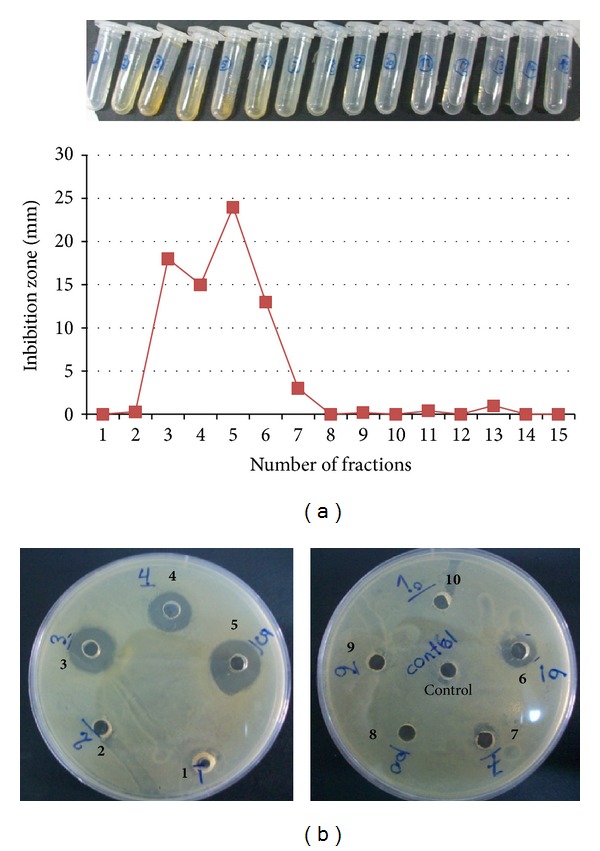
(a) Antibacterial activity of fractions numbers 1–15 of* Pseudonocardia* sp. JB05 extract. (b) Inhibition clear halo zone of* S. aureus* around the antibacterial fractions numbers 3, 4, 5, and 6 on agar plates.

Antimicrobial purification was performed for* Pseudonocardia* sp. JB05 extract because of its highest inhibition zone against* S. aureus*. The antibacterial supernatant was vacuum-evaporated to dryness and then extracted with dH_2_O and/or organic solvents (n-hexane and/or ethyl acetate). Our results showed that the dH_2_O extract was the most active extract against* S. aureus*, compared to those extracted using organic solvents. As an appropriate polar solvent, dH_2_O was used to extract the compound before being compared with the one from the organic solvent [18]. The dH_2_O extract was fractionated according to the following procedure: a total of 15 fractions were separated through a silica gel column (Figure 3(a)). When evaluated by an inhibition test, fractions numbers 3, 4, 5, and 6 indicated a clear inhibition zone (Figure 3(b)). They were pooled and loaded on G25 column chromatography and, after fractionation, fraction number 5 showed the largest clear zone against* S. aureus*.

Thin layer chromatography analysis revealed a blue-green band by using the anthrone reagent with *R*_*f*_ value of 0.8 which indicates the presence of carbohydrates in the purified antibacterial compound.

**Figure 4:**
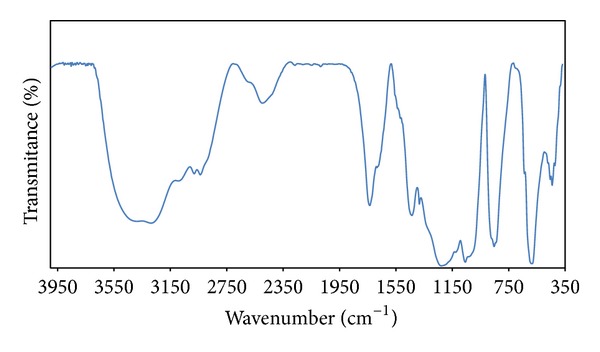
FT-IR analysis of the antibacterial compound isolated from* Pseudonocardia* sp. JB05.

Finally, an IR spectrum was obtained on a Bruker tensor 27 Fourier transform infrared spectroscopy (FT-IR) instrument. Accordingly, the FT-IR spectrum of dH_2_O extracts of* Pseudonocardia* sp. JB05 exhibited absorption around 3410 cm^−1^, which indicates hydroxyl groups, while the absorption at 2800–2915 cm^−1^ and at 1600 cm^−1^ indicates the presence of hydrocarbon skeleton and a double bond of polygenic compounds, respectively (Figure 4). Almost similar trend was observed in the FT-IR spectrum of ethyl acetate extract of* Streptomyces albidoflavus *PU23 [21]. The spectrum exhibited absorption bands at 3296 and 1031.8 cm^−1^, which is indicator of hydroxyl groups, while absorption at 1639 cm^−1^ indicates the presence of double bonds.

### 3.5. Protein Assay

Bradford analysis [14] using a standard curve showed that the amount of protein in the antibacterial active fractions was 0.0011 *μ*g/*μ*L.

### 3.6. Stability of Antibacterial Compound

**Figure 5:**
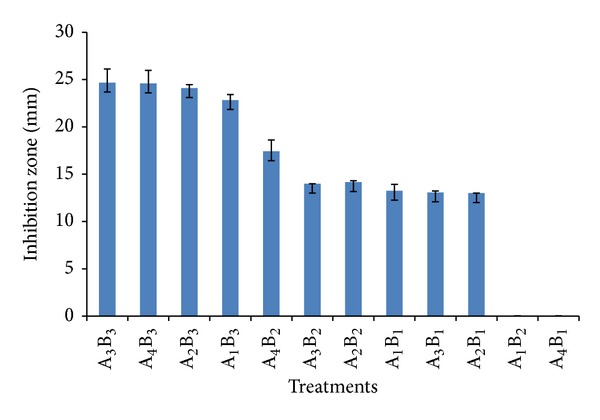
Effect of proteinase K, surfactant (10% SDS), and boiling on the antibacterial activity of four antibacterial compounds against* S. aureus*. A_1_:* Streptomyces *sp. JB07 (No. 012-1), A_2_:* Pseudonocardia* sp. JB06 (No. 012-2), A_3_:* Pseudonocardia* sp. JB05 (No. 010-31), A_4_:* Pseudonocardia* sp. JB02 (No. 025-26) and B_1_: proteinase K, B_2_: boiling, B_3_: 10% SDS.

The effects of proteinase K, surfactant (10% SDS), and boiling on the antibacterial activity of four antibacterial extracts against* S. aureus* were examined. The results showed that none of these above mentioned conditions had any significant effect on the antibacterial activity of the extract of bacterium number A_3_ (*Pseudonocardia* sp. JB05) (Table 1) (Figure 5). Also, autoclaving had no effect on antibacterial activity of this bacterium (Figure 5). MIC of* Pseudonocardia* sp. JB05 antibacterial compound was found to be 40 AU mL^−1^ against* S. aureus*.

## 4. Conclusion

*S. aureus* causes minor diseases, including life-threatening diseases. Some species of this bacterium are resistant to available antibiotics, such as beta-lactam antibiotics. Therefore, a new antibacterial compound is necessary to control the activity of this pathogenic bacterium. The present study was undertaken to evaluate the beneficial antibacterial effect of* Pseudonocardia* sp. JB05 on some pathogenic bacteria, especially* S. aureus.*

Different indigenous bacterial strains were isolated from alkaline soils of Hoze-Soltan, Qom, Iran, and compared for their ability to produce antibacterial compounds. Our results indicate that the strain* Pseudonocardia *sp. JB05 is the most effective candidate because of the antibacterial activity presented. The determination of JB05 antibacterial compounds indicated a minimum inhibitory concentration (MIC) of 40 AU mL^−1^ against* Staphylococcus aureus.*

The results also demonstrated that antibacterial activity by* Pseudonocardia *sp. JB05 was not influenced by the presence of surfactant (10% SDS), proteinase K, and boiling.

Thin layer chromatography, using anthrone reagent, showed the presence of carbohydrates in the purified antibacterial compound. Also, FT-IR spectrum of the active compound illustrated hydroxyl groups, hydrocarbon skeleton, and double bond of polygenic compounds in its structure. To the best of our knowledge, this is the first report describing the efficient antibacterial activity by a local strain of* Pseudonocardia. *The results presented in this work, although at the initial stage in bioactive product characterization, will possibly contribute toward the* Pseudonocardia *scale-up for the production and identification of the antibacterial compounds.

## Figures and Tables

**Table 1:** Morphological and biochemical characteristics and antibacterial activity of *Pseudonocardia* sp. JB05.

Properties	*Pseudonocardia *sp. JB05
Morphological characteristics	
Sporophor morphology	Straight
Color of aerial mycelium	Brown
Color of substrate mycelium	Reddish brown
Spore mass	Brown
Biochemical characteristics	
Gram staining	+
H_2_S production	−
Nitrate reduction	−
Urease	+
Catalase	++
Chitinase	−
Starch hydrolysis	+
Melanin production	−
Antibacterial effect on indicator strains	
* Staphylococcus aureus *	++
* Bacillus subtilis *	±
* Pseudomonas aeroginosa *	−
* Klebsiella *sp.	−
* Xanthomonas citri *	−
* Acinetobacter *sp.	−
* Escherichia coli *	−
* Bacillus pusteurii *	+

++: high activity; +: positive effect; −: negative effect.

++ = >20 mm, + = 10–20 mm, ± = 1–10 mm, − = 0 mm, and N = non determined.
